# Reconsidering functional redundancy in biodiversity research

**DOI:** 10.1038/s44185-023-00015-5

**Published:** 2023-04-27

**Authors:** Nico Eisenhauer, Jes Hines, Fernando T. Maestre, Matthias C. Rillig

**Affiliations:** 1 German Centre for Integrative Biodiversity Research (iDiv) Halle-Jena-Leipzig, Leipzig, Germany; 2 Institute of Biology, Leipzig University, Leipzig, Germany; 3 Instituto Multidisciplinar para el Estudio del Medio “Ramón Margalef”, Universidad de Alicante, Alicante, Spain; 4 Departamento de Ecología, Universidad de Alicante, Alicante, Spain; 5 Freie Universität Berlin, Institute of Biology, Berlin, Germany; 6 Berlin-Brandenburg Institute of Advanced Biodiversity Research (BBIB), Berlin, Germany

**Keywords:** Ecology, Ecology, Environmental sciences

## Abstract

A key question in ecological research is whether biodiversity is important for ecosystem functioning. After approximately three decades of empirical studies on this topic, it is clear that biodiversity promotes the magnitude and stability of ecosystem functioning. However, the majority of early biodiversity-ecosystem functioning (BEF) experiments concluded that there is a saturating relationship between biodiversity and ecosystem functioning, seemingly supporting the ‘*redundancy hypothesis*’ of biodiversity. This hypothesis may suggest that many species can be lost from an ecosystem before any changes in functioning can be detected under the current environmental conditions. Here, we argue that the term functional redundancy (1) may have been overused from an ecological perspective and (2) can be dangerous and misleading in scientific communication. Rather, we propose to use the term ‘*functional similarity*’, which better highlights the unique contributions of all coexisting species to ecosystem functioning, gradients in niche overlap and has a less negative connotation. In a world where increasing anthropogenic stressors are accelerating biodiversity change and loss and thus threatening ecosystem integrity, important political and societal decisions must be taken to combat the joint climate and biodiversity crisis. We should therefore reconsider and carefully choose terminology in biodiversity science for value-neutral communication.

## Background

A key question in ecological research is whether biodiversity is important for ecosystem functioning. The hundreds of studies exploring this relationship^1,2^ provide convincing evidence for a positive effect of biodiversity on ecosystem functioning (BEF), despite some context-dependency in the strength of the effect^3^. This work highlights the functional differences among species, *i.e. functional complementarity*, as a major mechanism behind the positive BEF relationship^2–4^. Functional complementarity entails dissimilarities among co-existing species in resource partitioning, abiotic facilitation, and biotic feedbacks^5^. However, the majority of early BEF experiments concluded that there is a saturating relationship between biodiversity and ecosystem functioning^1,6^, seemingly supporting the ‘*redundancy hypothesis*’ (or ‘*rivet-redundancy hypothesis*’) of biodiversity^7–10^. This hypothesis may suggest that many species can be lost from an ecosystem before any changes in functioning can be detected under the current environmental conditions^11^. As a consequence, the term *redundancy* has been frequently used to describe a situation where different coexisting species seem to fulfill the same ecological role and are exchangeable. For instance, soil microbiologists are often referring to the functional redundancy of microorganisms for ecosystem functioning^12–15^.

**Fig. 1:**
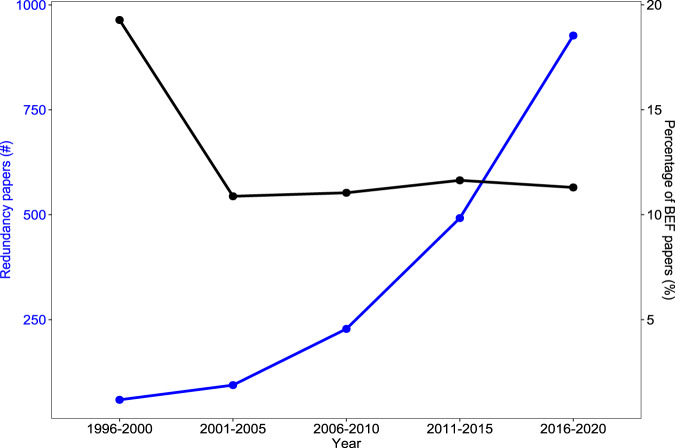
The number of biodiversity papers using the term ‘redundancy’ relative to the number of Biodiversity-Ecosystem Functioning (BEF) papers. The data stems from Web of Science searches for Biodiv* AND Redundan*, and Biodiv* AND Ecosystem Function* on 6 September 2022. Five-year intervals are illustrated from 1996–2020. Although absolute numbers of publications are not comparable between 2021–2022 and the 5-year intervals, the proportion of ‘*redundancy papers*’ was also at ~11% in these two years. The reader should note that we did not explore how the term redundancy was used in this literature, as this was beyond the scope of our *Comment*.

Already more than two decades ago, a comment by Ehrlich and Walker^11^ highlighted the nuances of the *redundancy hypothesis* by stating that (1) species redundancy is a critical property that contributes to ecosystem resilience; and that (2) a functional group approach may be useful to identify very important species to conserve. They also highlighted that this does not mean that we can afford losing any species from an ecosystem though. Moreover, a conceptual paper by Loreau^10^ used a classical Lotka-Volterra model to show that stable coexistence is incompatible with functional redundancy under equilibrium conditions, as stable coexistence requires differences between species and, thus, functional complementarity. Nevertheless, supported by some empirical evidence^6^, the concept of *functional redundancy* has been widely used in the ecological literature until today (Fig. 1). In this Comment, we argue that the term *functional redundancy* (1) may have been overused from an ecological perspective and (2) can be dangerous and misleading in science communication. Instead, we propose to reconsider using the term *functional redundancy* and use the term *functional similarity* to offer a gradient-based alternative for value-neutral communication.

### Revisiting ecological theory and evidence for functional redundancy

Ecological theory predicts that coexisting species should differ in their traits or environmentally-mediated relative fitness, because otherwise there would be competitive exclusion^10,16–18^. How does this relate to the concept of *functional redundancy* of some coexisting species? Loreau^10^ concluded that transient non-equilibrium coexistence can obscure functional complementarity and allow for functional redundancy only in the short term^19^. However, for long-term coexistence, species must show different responses or fitness differences to environmental fluctuations (*temporal niche differentiation*) and/or to spatial environmental heterogeneity (*spatial niche differentiation*). In contrast to this theory, by summarizing the available data at that time, Cardinale and colleagues^6^ found overwhelming support for the *redundancy hypothesis* across many terrestrial and aquatic biodiversity experiments. However, their synthesis mainly included short-term experiments, and more recent long-term experiments have shown increasing biodiversity effects over time^20–23^ up to almost linear relationships between biodiversity and ecosystem functioning^21,23^. This means that the strength of biodiversity effects on various ecosystem functions in grassland biodiversity experiments increases from neutral (*e.g*. Eisenhauer et al.^24^ for soil microbial biomass) or weakly positive (*e.g*. Reich et al.^21^ for plant biomass production) after a couple of years to strongly positive after >4 years of the respective experiment. Stronger long-term BEF relationships have been shown to emerge from increasing functional diversity^25^ and complementarity effects^21,23^ over time. Moreover, all of these conclusions were primarily based on one single function (mostly primary productivity), while a global synthesis revealed that different plant species promoted different functions, during different years, at different places, and under different environmental change scenarios^26^. The authors of this study stated that although species may appear functionally redundant when one function is considered under one set of environmental conditions, many species are needed to maintain multiple functions at multiple times and places in a changing world^26^. This finding is more in line with the *singular hypothesis* of biodiversity, stating that each coexisting species has a unique effect on ecosystem functioning^24^ because of the predicted spatial and temporal niche differentiation^10^.

Taking a trait-based perspective, Pillar et al.^27^ stated that “*functional redundancy is dependent on the traits that are used for the computation of functional diversity*” and that “*it is assumed that the traits are functional for the ecosystem process being considered, and thus the functional redundancy that is measured refers to redundancy for the process in hand*.” They conclude that “*the question ‘redundancy for what?’ should always be asked*”. Accordingly, some authors have defined redundancy as the ‘*functional dissimilarity between those species with similar effect on ecosystem processes*’ (reviewed in de Bello et al.^28^), stressing gradients in functional similarity. A related concept is “*response diversity*”^29^, since species may be functionally similar for the ecosystem effect under consideration but may be different in their responses (based on *response traits*) to environmental factors. This highlights the difficulties in distinguishing *response traits* and *effect traits* to properly assess *functional redundancy* as well as the significance of gradients in response diversity as key concept for understanding the role of biodiversity in ecosystem functioning and stability.

Assessing spatial and temporal niche differentiation may be particularly challenging for soil microorganisms. As a consequence, the immense diversity of microbial taxa makes an *a priori* assumption of *functional redundancy* more likely as a working hypothesis^30^. However, the concept of competitive exclusion has also been documented for microorganisms^31,32^, and relative fitness differences have been suggested as a relevant mechanism explaining the coexistence of ecologically similar soil microorganisms^33^. In this context, *functional redundancy* was defined by Allison & Martiny^12^ as the “*ability of one microbial taxon to carry out a process at the same rate as another under the same environmental conditions*”. Accordingly, many studies focusing on broad functions like nutrient cycling and decomposition^30,34,35^ concluded that there is high *functional redundancy* in the microbial world^13^. Again, we argue that the context-dependent effects described above may play a crucial role, since researchers have mostly looked at snapshot assessments of microbial diversity and broad functions, mostly missing information on relevant subprocesses^30,36,37^ and on which taxa are actually really active at that point in time^38,39^. While microbial ecologists are relating the diversity and composition of microbial communities to a wealth of ecosystem functions (*e.g*. decomposition, nitrification, denitrification, methanotrophy, methanogenesis) using complex (functional, genomic, phylogenetic) approaches that shed light on the relationship between biodiversity and ecosystem functioning, there is the risk of a mismatch in scope in such endeavors, since the processes we commonly measure (at the ecosystem scale, such as decomposition) are not easily matched to the microbial or gene expression or enzyme production scale. This is because ecosystem processes are a consequence of many different component processes^37^. This scale and process mismatch may be more pronounced for microbes (with many undescribed taxa) than for other groups of well-studied taxa like plants. Even if we only look at a single soil core typically taken to explore soil microbial diversity and functions, we need to consider that microorganisms live in and on top of tiny soil aggregates that represent different ‘universes’ of species assemblages that will never interact with each other^40,41^. So, when mixing these universes, we also need to adapt our interpretation of BEF relationships and the contributions of individual taxa. This awareness has important implications for biogeochemical processes and the role of soil microbial community composition^30^. Taken together, we believe that the environmental conditions are exceptionally challenging to determine at the scales we would need to measure them to adequately test for *functional redundancy*.

### Dangerous language in communication and outreach

Another important issue with the communication of scientific findings is the public perception of biodiversity that results from consuming the scientific literature. The term *‘redundancy’* has a negative connotation in the sense that it suggests that we can easily lose species without any detrimental ecosystem effects, since the word ‘redundant’ in everyday use is equivalent to ‘expendable’ or ‘unnecessary’. As such, this term can be problematic for value-neutral, objective communication about the functions of biodiversity: the word may easily be misunderstood without further context and thus requires additional explanation and clarification. This potential communication problem is exacerbated at the science-policy interface, where the very conservation of biodiversity is at stake. Nevertheless, language influences and mirrors our thinking as scientists, and thus we should be careful with the terms we use, not just for outreach beyond science, but also when communicating our research findings within our own community.

### Conclusion and way forward

We suggest critically reconsidering the term *redundancy* in biodiversity research and any related science communication with regards to the role of species in ecosystems. An alternative expression would be referring to the ‘*functionally similarity*’ (or dissimilarity) of species. *Functional similarity* describes a gradient in potential niche overlap among species or communities that has been commonly used for variety of taxa, ranging from microorganisms to animals^12,42–45^. When referring to *functional similarity*, it will also be helpful to always clearly define the function under consideration as well as the environmental parameter space and context^46^, to avoid blanket statements about general functional similarity (*e.g*. “*there is [high] functional similarity under certain environmental conditions for the decomposition of lignin*”). Moreover, we believe that the term *functionality similarity* helps highlighting a gradient-based concept, rather than any binary description of niche overlap. In a world where increasing anthropogenic stressors are accelerating biodiversity change and loss and thus threatening ecosystem integrity, important political and societal decisions must be taken to combat the joint climate and biodiversity crisis^47^. We should therefore reconsider and carefully choose terminology in biodiversity science for value-neutral communication.

## Data Availability

Not applicable.

## Code availability

The search term for our literature search is specified in the legend of Fig. 1.
